# Lying flat to play on smartphone excessively: the role of self-esteem

**DOI:** 10.3389/fpsyg.2025.1516869

**Published:** 2025-02-11

**Authors:** Shuhua Zhu, Lishen Wang, Yulu Gan

**Affiliations:** ^1^Department of Development and Planning, Zhejiang Ocean University, Zhoushan, China; ^2^School of Education Science, Shaoguan University, Shaoguan, China; ^3^Mental Health Education Centre, Zhejiang International Maritime College, Zhoushan, China

**Keywords:** lying flat, involution, problematic smartphone use, self-esteem, mediating role

## Abstract

**Introduction:**

With the rapid development of their countries, Chinese young people today face intense stress. Some cope by striving for high-quality resources (involution), while others adopt a passive approach, lowering their desires (lying flat). This study explores the relationship between lying flat and problematic smartphone use (PSU), focusing on the role of self-esteem.

**Methods:**

We conducted three studies (*N* = 886) using both cross-sectional (Study 1) and experimental (Study 2 and Study 3) designs to examine the correlational and causal links between lying flat, self-esteem, and PSU.

**Results:**

The studies demonstrated a significant relationship between lying flat and PSU, with self-esteem serving as a mediator. Additionally, Study 3 showed a causal effect of self-esteem on smartphone use desire.

**Discussion:**

These findings provide insight into why and how lying flat affects self-esteem and smartphone use behaviors and desires, highlighting the psychological mechanisms behind the trends observed in Chinese youth.

## Introduction

Rapid development of economy and technology brings wealth and convenience, but also evokes social competition and injustice. Chinese people, especially the youngsters, are under intense stress, such as excessive social competition, unsuitable school education, and overtime work (Hsu, 2022). In response to such collective stress, two strategies became popular (He et al., 2024; Ou, 2023; Xiong, 2022; Zhou, 2022). One is facing the challenge directly and invest more time and effort to compete for high-quality resources, as the buzzword “Neijuan (内卷)” or involution goes. The other is intentionally reducing one’s expectations and withdraw from competition so as to alleviate stress, as the buzzword “Tangping (躺平)” or lying flat goes. In such an digital era, escaping from real society and entering the digital world is easily. A smart cell phone can satisfy many unmet needs in social life. That’s why an increasing number of youngsters became addicted to digital device (e.g., smartphone; Olson et al., 2022). Therefore, it is plausible to posit that lying flat (vs. involution) may cause problematic smartphone use (PSU). In the present work, we systematically test such a proposal as well as the potential underlying mechanism.

### Lying flat and PSU

Lying flat, a buzzword appeared on the list of the top ten buzzwords of the year 2021 in China, has become another “life philosophy” for the generation of young people (Ou, 2023; Xiong, 2022). There are also similar words in other countries describing youth withdrawal or “stagnant youth,” for example, British NEET, the American Boomerang Kids, the Japanese hikikomori (low desire group), and the Korean social animal spirit (Lu et al., 2023; Zhou, 2023). Lying flat seems to be a global phenomenon, the proportion in the population aged between 15 and 29 who choose to lie flat is more than 10%, with the United States for 14.5%, the United Kingdom for 10.6%, France for 13.4%, Italy for 22.9%, and Mexico for 19.5% (OECD, 2024). “Lying flat” philosophy mainly refers to withdrawal from a world full of competition and pressure (Ou, 2023; Xiong, 2022). That is, people choose “lying flat” so as to avoid fierce competition, especially when the environment is unfair making individuals feel hardly get returns of equal value through their personal effort (Hsu, 2022; Ma and Wang, 2022; Qin and Dai, 2022).

In contrast, people may invest increasing time and effort to compete for high-quality resources when facing fierce social competition (Yi et al., 2022). Such a strategy is termed “involution (neijuan, 内卷)” in China, another buzzword referring to irrational and involuntary internal competition for high-quality resources. Given that Confucian culture highly stress the efforts of individuals, involution is also a popular lifestyle (Hsu, 2022).

These two buzzwords reflect the current Chinese youth strategy or mentality in response to the fierce social competition and pressure. Adopting Evolutionary Game Theory, He et al. (2024) showed that players would endorse different strategies facing intense competition for resources in an Involution-Cooperation-Lying Flat Game, reflecting how individuals do in the real social competition.

Furthermore, strategies like involution and lying flat also influence individuals’ psychology and behavior. Although involution may not lead to satisfactory outcomes and even exert detrimental influence on individuals, it may not be necessary harmful for individuals (Yi et al., 2022). For example, empirical research showed that achievement-motivated involution was negatively related with anxiety (Yi et al., 2022). Because spending time and effort on valued activities is helpful in enhancing sense of control and combating boredom, and may also promote performance to some extent (e.g., in education; Yang et al., 2023). Individuals who adopt involution strategy spend more time and effort on their valued activities, rather than on their digital device (e.g., smartphone). Therefore, they are less likely to use smartphones and other digital device excessively.

However, individuals who adopt lying flat strategy are more likely to be addicted to smartphones and other digital device. The logic of lying flat is: If I do not have too much desire and avoid working in high-pressure social settings, I can avoid the feelings of hopelessness and worthlessness, and regain the sense of self-worth (Song and Bie, 2022). Unfortunately, lying flat during the best years of life stage may aggravate the situation, instead of leading to sense of safe, self-worth, and happiness (Song and Bie, 2022). As previous work suggested, doing nothing or inactivity will increase negative thoughts and make people unhappier (Yang and Hsee, 2019), and even lead to poor mental health (Lu et al., 2024). If the youngsters lost their ambition and motivation, they will probably turn to the virtual world through the most convenient tools (e.g., smartphones; Deng et al., 2022). Playing on smartphone is effortless, and many unmet needs could be satisfied (Billieux, 2012; Olson et al., 2022). Therefore, individuals who choose lying flat are also likely to use smartphones problematically.

### The role of self-esteem

We further propose that self-esteem, the overall sense of worthiness to be a person (Rosenberg, 1965), can mediate the link between lying flat and problematic smartphone use. On one hand, lying flat dampens self-esteem. Lying flat often happens after one’s failure in real life, when they usually self-deprecate (Song and Bie, 2022). Given that most youngsters choose lying flat due to lack of meaning and value in real life, they seek to regain the sense of meaning and value through lowering their expectations. However, the lowered expectations or desires lead them to do little to improve themselves, which may lower, instead of regaining, self-esteem (Zheng et al., 2023). Additionally, lying flat is not acceptable in Confucian culture that emphasizes individuals’ effort, which may make individuals facing another form of social pressure and have less self-worth (Hsu, 2022).

On the other hand, low self-esteem is a significant pathway leading to problematic smartphone use, according to the pathway model of problematic mobile phone use (Billieux, 2012). Individuals with low self-esteem cannot evaluate themselves and the external world objectively and accurately, resulting in low self-acceptance and low expectations for the real world and turn to seek respect in virtual world (Li et al., 2019). Additionally, individuals with low self-esteem usually have interpersonal problems in real social life and socializing with others online is a better way to fulfill their social needs or compensate for fear of social loss (Bianchi and Phillips, 2005; You et al., 2019). Rai et al. (2019) showed that people with low self-esteem prefer indirect communication. And a great deal of studies demonstrated a significant relationship between low self-esteem and problematic smartphone use (for meta-analyses, see: Casale et al., 2022; Liang et al., 2024), although most of such evidence was correlational.

### Overview

Three studies were conducted to test our prediction that lying flat is linked with problematic smartphone use, correlationally and causally, through (low) self-esteem. In Study 1, we examined the correlation among lying flat, self-esteem and problematic smartphone use (PSU) at the trait level, by surveying a sample of Chinese college students. Studies 2 and 3 aimed to test the causal link of these three constructs at the state level. In Study 2, we manipulated lying flat (vs. involution) intention by asking them to imagine to do so in the future and assessed state self-esteem and smartphone use desire. Desire for using smartphone is a useful index to assess problematic smartphone use in experiments (Zhu, 2024). In Study 3, we manipulated the mediator (i.e., self-esteem) and assessed smartphone use desire (the dependent variable), following an experimental procedure to test the causality of mediator and dependent variable (Ge, 2023; Spencer et al., 2005). All the three studies were approved by the institutional review board of the first author’s institution on 15th, November, 2023. All the data and code for the present work can be found at OSF.^1^

## Study 1: lying flat predicts problematic smartphone use via self-esteem

Study 1 was conducted to test whether lying flat was positively associated with problematic smartphone use, as well as the putative mediating role of self-esteem, using cross-sectional design.

### Method

#### Participants

As suggested by Schönbrodt and Perugini (2013), at least 250 participants were needed to obtain a stable correlation. We recruited 495 university students (282 men, 213 women) from psychological courses, whose age ranged from 16 to 20 years (*M* = 18.36, *M* denotes the mean value of a given variable in the sample. *M* = (X1 + X2 + … + Xn)/n, *n* is the total number of participants. *SD* = 0.52, *SD* denotes the standard deviation, a measure of the spread or dispersion of values around the mean. *SD* = \sqrt{\frac{\sum (xi − *M*)^2}{n}}, xi represents each individual value, *n* is the total number of participants). Participants were asked to complete measures of interest via wenjuanxing, a platform similar qualtrics, after giving their informed consent. Participants of the present study and the following two read a detailed document describing our research purpose and their rights and reimbursement and signed the formal consent form, before they began to complete the research tasks. All participants completed our study were reimbursed with partial course credit.

#### Measures

##### Lying flat

We used the reliable and valid Lying Flat Tendency scale to assess participants’ general tendency to lying flat (Lu et al., 2023)^2^. The scale consists of six items (a sample item: “*I do not have any goals and pursuits for life and study, and I am really lying flat in action*”). Participants were asked to rate each item on a four point scale (1 = *very inconsistent*, 4 = *very consistent*). We summed and averaged the ratings for each participant to form a general indicator of lying flat tendency (*M* = 2.12, *SD* = 0.58, *ɑ* = 0.84, *ɑ* denotes the Cronbach’s alpha, a measure of internal consistency or reliability of a scale. *ɑ* = (K / (K−1)) * (1 − (Σ*Si*^2^ / *Sx*^2^)), K is the number of items on the scale, Si^2^ is the variance of the ith item, Sx^2^ is the variance of the total score).

##### Problematic smartphone use

We adopted the Chinese version of the Smartphone Addiction Scale (short form) to capture problematic smartphone use (Luk et al., 2018). Its Chinese version is reliable and valid (Luk et al., 2018; Zhu, 2024). Participants indicated their agreement with each of the ten items on a 6-point Likert scale (1 = *strongly disagree*, 6 = *strongly agree*; a sample item:“*Missing planned work due to smartphone use*”). We summed and averaged the ratings for each participant to generate a general index of problematic smartphone use (*M* = 3.54, *SD* = 0.90, *ɑ* = 0.88).

##### Self-esteem

The Chinese version of Rosenberg (1965) Self-Esteem Scale (for Chinese version, see Ji and Yu, 1999) was used to assess participants’ self-esteem. This is a widely used scale capturing self-esteem. Participants indicated their agreement with each item on a 4-point scale (1 = *strongly disagree*, 4 = *strongly agree*; *M* = 2.95, *SD* = 0.50, *ɑ* = 0.86).

### Results and discussion

As predicted, lying flat was positively associated with problematic smartphone use, *r* (495) = 0.45, *r* denotes the correlation coefficient, which measures the strength and direction of the linear relationship between two variables. *r* = (*Σ*(Xi − X̄)(Yi − Ȳ)) / (sqrt(*Σ*(Xi − X̄)^2^) * sqrt(Σ(Yi − Ȳ)^2^)), xi and yi are the individual values of the two variables, X̄ and Ȳ are the means of the two variables. *p* < 0.001, *p* denotes the probability of obtaining results at least as extreme as the results actually observed, assuming that the null hypothesis is correct, while negatively associated with self-esteem, *r* (495) = −0.44, *p* < 0.001. Self-esteem was also negatively associated with problematic smartphone use, *r* (495) = −0.29, *p* < 0.001.

**Figure 1:**
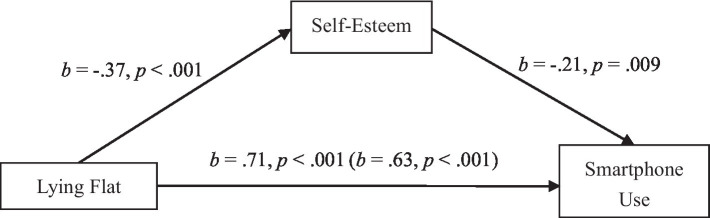
Self-esteem mediates the link between lying flat and problematic smartphone use in study 1.

Given the significant associations among the three variables, we can proceed to examine the mediating role of self-esteem in the relationship between lying flat and problematic smartphone use. Following the mediation analysis procedure (Hayes, 2018), we took lying flat, self-esteem, and problematic smartphone use as independent, mediational, and dependent variables, respectively. In addition, we also included age and gender as covariates in the mediation model. The bias-corrected Bootstrapping with 5,000 resamples showed that the indirect effect did not contain zero, supporting the significant mediating role of self-esteem, *F* (4, 490) = 33.61, *F* denotes the *F*-statistic, which is used to determine whether the overall regression model is a good fit for the data. *F* = Model Mean Square (MSM)/Model Mean Square (MSM). *R*^2^ = 0.22, *R*^2^ denotes the proportion of variance in the dependent variable that is explained by the independent variables in the model. *R^2^* = 1− (*Σ*(yi − ŷi)^2) / Σ(yi − ȳ)^2, yi is the observed value of the dependent variable, ŷi is the predicted value from the model,ȳ is the mean of the observed values. *p* < 0.001, *SE* = 0.04, *SE* denotes the standard error, which is a measure of the variability or precision of an estimate, such as a regression coefficient or a mean. *SE* = SD /√n, *SD* is the standard deviation, *n* is the number of data points. indirect effect = 0.08, indirect effect denotes the portion of the total effect of one variable on another that is mediated through a third variable (in this case, self-esteem mediating the relationship between lying flat and problematic smartphone use). Indirect Effect = (a × b), a is the effect of the independent variable on the mediator, b is the effect of the mediator on the dependent variable. 95% CI [0.01, 0.15], 95% CI provides a range of values within which the true population parameter is expected to lie with 95% confidence. 95%CI = X̄±1.96 × SE, X̄ is the sample mean, *SE* is the standard error of the estimate (Figure 1). Therefore, the Study 1 findings supported our hypothesis that self-esteem played a mediating role in the relationship between lying flat and problematic smartphone use.

## Study 2: primed lying flat promotes desire for using smartphone via decreased self-esteem

Study 1 showed that there was a significant relationship between lying flat and problematic smartphone use, with self-esteem as a mediator. Study 2 aimed to test the causality of these associations, by manipulating lying flat and assessing desire for using smartphone and state self-esteem. Desire for using smartphone was used to act as a state indicator of problematic smartphone use as Zhu (2024) did, so that we can experimentally explore the factors influencing problematic smartphone use. We expected that participants in the lying flat (vs. involution) condition would report lower self-esteem and higher desire for using smartphone, and the effect of condition on desire for using smartphone was mediated by lowered self-esteem.

### Method

#### Participants

We determined our sample size for proposed mediation model using Monte Carlo power analysis for indirect effects application (Schoemann et al., 2017). Assuming medium inter-correlations among the manipulation (lying flat vs. involution), mediator (i.e., state self-esteem), and dependent variable (i.e., smartphone use desire) of *r* = 0.30 (*SD* = 0.10). To obtain power 0.80 at *α* = 0.05, we needed at least 160 participants. We recruited 192 Chinese participants (123 women, 69 men) via Credamo, a Chinese online platform similar to Qualtrics, in exchange of 10 Yuan (≈$1.50). Participants’ age ranged from 19 to 57 years (*M* = 29.99, *SD* = 8.04).

#### Procedure

Participants were randomly assigned to the lying flat (*n* = 97) or involution (*n* = 95) condition. Participants in lying flat (vs. involution) condition were asked to read a brief description of the buzzword lying flat (vs. involution), and imagine what they would do if they decide to endorse the lying flat (vs. involution) mentality. Below are detailed instructions:

*The buzzword lying flat (vs. involution) describes a “life philosophy or mentality” endorsed by individuals who intentionally reduce their expectations and withdraw from competing for high-quality resources (vs. face the challenge directly and invest more time and effort to compete for high-quality resources). Many people choose lying flat (vs. involution) mentality in modern society. Please imagine what you would do if you endorse such a mentality, and write down at least 100 words to describe your typical lying flat (vs. involution) action.*

Next, participants were asked to complete measures below according to their current feelings evoked by the mentality they imagined to endorse.

The first measure was a state version of lying flat, acting as a manipulation check. We intentionally adapted the six items of Lying Flat Tendency scale (Lu et al., 2023) to capture participants’ lying flat intention. A sample item was: “*Right now, I do not have any goals and pursuits for life and study, and I’m going to lie flat in action*”. Participants were asked to rate each item on a four point scale (1 = *strongly disagree*, 4 = *strongly agree*). The average score on the six items for each participant was calculated to form a general indicator of lying flat intention (*M* = 2.18, *SD* = 0.73, *ɑ* = 0.88).

The second measure was the state version of Rosenberg (1965) Self-Esteem Scale (for Chinese version, see Ji and Yu, 1999; Yang et al., 2024). Each item was preceded by the stem “Right now.” Participants indicated their agreement with each item on a 4-point scale (1 = *strongly disagree*, 4 = *strongly agree*; *M* = 3.19, *SD* = 0.61, ɑ = 0.92).

The third measure was the smartphone use desire scale, developed by Zhu (2024). Participants indicated their agreement with the four items on a 7-point likert scale (1 = *strongly disagree*, 7 = *strongly agree;* a sample item:*“I have a desire for using my smartphone right now”*). We summed and averaged each participant’s ratings on the items, high score indicating strong desire for using smartphone (*M* = 3.92, *SD* = 1.62, *ɑ* = 0.93).

### Results and discussion

#### Manipulation check

An independent sample *t*-test showed that participants in the lying flat condition (*M* = 2.53, *SD* = 0.74) reported significantly higher score on lying flat intention items than those in the involution condition (*M* = 1.82, *SD* = 0.51), *t* (190) = 7.72, *t* is used in hypothesis testing to determine whether the difference between two groups is statistically significant. *t* = (X1–X2) / sqrt((s1^2 / n1) + (s2^2 / n2)), X1 and X2 are the means of the two groups,s1 and s2 are the standard deviations of the two groups,n1 and n2 are the sample sizes of the two groups. *p* < 0.001, Cohen’s *d* = 1.11, *Cohen’s d* measures the size of the difference between two groups in terms of standard deviations. It is often used to interpret the practical significance of the results. *d* = (M1−M2) / SD, M1 and M2 are the means of the two groups, SD *is* the standard deviation, 95% CI [0.82, 1.40]. Therefore, our manipulation procedure to manipulate lying flat mentality was successful.

#### Self-esteem

As predicted, participants in the lying flat condition (*M* = 2.99, *SD* = 0.73) reported significantly lower state self-esteem than those in the involution condition (*M* = 3.38, *SD* = 0.34), *t* (190) = −4.69, *p* < 0.001, Cohen’s *d* = −0.68, 95% CI [−0.96, −0.39].

#### Smartphone use desire

Also as predicted, participants in the lying flat condition (*M* = 4.47, *SD* = 1.67) reported significantly more intense desire for using smartphone than those in the involution condition (*M* = 3.35, *SD* = 1.36), *t* (190) = 5.11, *p* < 0.001, Cohen’s *d* = 0.74, 95% CI [0.45, 1.02].

#### Mediation analysis

**Figure 2:**
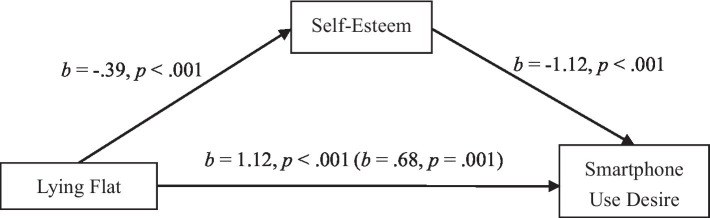
Self-esteem mediates the effect of lying flat on smartphone use desire in study 2.

We proceeded to test the mediating role of state self-esteem in the effect of lying flat on desire for using smartphone. We entered the dummy-coded condition (Hayes, 2018; 1 = *lying flat condition*, 0 = *involution condition*) as independent variable, state self-esteem as mediator, smartphone use desire as dependent variable, respectively, in the mediation analysis. The results of bias-corrected bootstrapping with 5,000 resamples showed that the indirect effect via self-esteem excluded zero, *F* (2, 189) = 36.66, R^2^ = 0.28, *p* < 0.001, *SE* = 0.11, indirect effect = 0.44, 95% CI [0.25, 0.67] (Figure 2)^3^. These findings suggested that decreased state self-esteem played a significant mediating role in the causal effect of lying flat on smartphone use desire.

## Study 3: the causal link between self-esteem and desire for using smartphone

Although Study 2 finding suggested there was a causal chain among lying flat, self-esteem, and smartphone use desire, it is inconclusive because we assessed self-esteem and smartphone use desire simultaneously. It is necessary to manipulate mediator and assess dependent variable, so as to test the causal chain of the mediation model (Ge, 2023; Spencer et al., 2005). Therefore, Study 3 was conducted to test the causal link between self-esteem and desire for using smartphone, by manipulating the mediator (i.e., self-esteem) of our hypothesized mediation model and assessing dependent variable (i.e., desire for using smartphone). We anticipated that participants in high (vs. low) self-esteem condition would report stronger desire of using smartphone.

### Method

#### Participants

We first determined the sample size needed to obtain a medium effect size for independent sample *t*-tests with *a priori* power analysis (G*Power 3.1; Faul et al., 2007). At least 102 participants were required under standard criteria (*α* = 0.05, *β* = 0.80). We recruited 199 participants (120 women, 79 men) via Credamo, who took part in our study in exchange of 5 Chinese Yuan (approximately 0.8 US dollars). The age of the participants ranged from 18 to 72 (*M* = 30.35, *SD* = 9.84).

#### Procedure

Participants were randomly assigned to the high (*n* = 100) or low (*n* = 99) self-esteem condition. A self-esteem manipulation procedure validated by Mahadevan et al. (2023) was adopted (see also Study 10 in Yang et al., 2024 work). Participants, in the high self-esteem condition, were instructed to think about ways in which they felt like they were a worthy person with many good qualities, and were satisfied with themselves. In contrast, participants in the low self-esteem condition were asked to think about ways in which they felt like a failure with few good qualities, and were dissatisfied with themselves. Next, participants were instructed to list three relevant keywords and describe the pertinent ways they thought about for at least 3 min. Finally, they were asked to complete the manipulation check questions and a measure assessing smartphone use desire.

#### Manipulation check

Three items that have been used in prior work to assess state self-esteem were used as manipulation check (Mahadevan et al., 2023; Robins et al., 2001). A sample item was “*Right now, I have high self-esteem*” (1 = *strongly disagree*, 7 = *strongly agree*). We summed and averaged ratings on these three items for each participant to form an index of state self-esteem (*M* = 5.07, *SD* = 1.25, *α* = 0.82).

#### Smartphone use desire

Identical to Study 2 (*M* = 3.73, *SD* = 1.39, *ɑ* = 0.91).

### Results and discussion

#### Manipulation check

An independent sample *t*-test showed that participants in the high self-esteem condition (*M* = 5.80, *SD* = 0.70) reported significantly higher score on state self-esteem items than those in the low self-esteem condition (*M* = 4.33, *SD* = 1.26), *t* (190) = 10.18, *p* < 0.001, Cohen’s *d* = 1.44, 95% CI [1.16, 1.72]. Therefore, our manipulation procedure was successful.

#### Smartphone use desire

As predicted, participants in the high self-esteem condition (*M* = 3.46, *SD* = 1.38) reported significantly less desire for using smartphone than those in low self-esteem condition (*M* = 4.01, *SD* = 1.34), *t* (190) = −2.84, *p* = 0.005, Cohen’s *d* = −0.40, 95% CI [−0.68, −0.12]. These findings confirmed the causal effect of self-esteem on decreasing desire for using smartphone.

## General discussion

With the rapid development of society and technology, youngsters need to cope with the challenge of fierce social competition and pressure. That’s why two buzzwords, lying flat and involution, corresponding to two coping strategies or mentality, become popular all over the internet (Deng et al., 2022). Lying flat can release individuals’ pressure in a short time at the cost of lowering their sense of self-worth and losing the chance to accomplish their improvement in real life. In the long run, however, people choose lying flat will feel tired, anxious, and constantly consume themselves, and even be addicted to the digital device (e.g., smartphones), as our work suggested.

Across three studies, we showed that lying flat was positively associated with and could facilitate problematic smartphone use (desire), being mediated by self-esteem. Specifically, cross-sectional Study 1 showed that lying flat could predict problematic smartphone use at the trait level. In experimental Study 2, participants who were asked to imagine they would adopt lying flat (vs. involution) mentality reported more intense desire to use smartphone, a critical indicator of problematic smartphone use as suggested by Zhu (2024). Both findings of Studies 1 and 2 supported the mediating role of self-esteem in the correlational and causal link between lying flat and smartphone use. Study 3 further indicated the causal effect of self-esteem on smartphone use desire, by manipulating participants’ state self-esteem.

## Implications

When people lose the desire to self-improve and tend to do nothing or little in their real life, they may also lose their self-worth and probably immerse themselves into the virtual world, for example, using smartphones problematically. Importantly, our findings are not limited to young samples, because the age of our samples ranged from 16 to 72 years, which suggests that people of all age may be affected by the lying flat mentality. Therefore, we argue that the social and technological development may affect people all over the age ranges. Such a negative mentality or subculture of lying flat is also harmful for the development of the society and the overall quality of people at the collective level. Besides, it may also influence people in other countries who have the similar mentality or subculture such as in America, England, Japan and South Korea (Lu, 2023; Zhou, 2023). Hence, lying flat deserves researchers’ further investigation and policy-makers’ attention.

As a mentality or self-defense strategy, lying flat can affect individuals self-systems and their interaction with digital device. Individuals who choose lying flat will not self-improve, losing their ambition and motivation. Psychologists could develop theories linking lying flat mentality with self-concept, as our work and others who have investigated lying flat and self (Zheng et al., 2023). Supporting Zheng et al. (2023), it is plausible to take lying flat as an opponent of self-improvement. Contrary to the purpose who endorse lying flat mentality, lying flat decreases rather than regains or compensate for one’s self-esteem.

In such a digital era, it is also vital for researchers to study the relationship between lying flat mentality and digital device use. Although digital device especially the smartphones brings enormous convenience and satisfies our needs, we should avoid using them excessively (Olson et al., 2022). Unfortunately, people with lying flat mentality and low self-esteem are more likely to use smartphones problematically. Our work highlights the significance of studying the influence of social and technological development on device dependence and self-esteem.

For policy-makers, more effort is needed to work out policies to make people see the hope and opportunity to self-improve during the rapid social development. Although we did not explore why people adopt lying flat mentality, it is important to realize the detrimental effect of such a mentality on self-esteem and behavioral addiction (problematic smartphone use). As previous studies suggested, if the system is fair and most people can achieve their goals through effort, people may not lie flat (Deng et al., 2022; Zheng et al., 2023). Additionally, more proper and effective measures are needed, as Lu et al. (2024) showed that watching inspirational video for 8 days could lower participants’ lying flat tendency. Smartphone is a useful tool, which can also be utilized to inspire the youth. Policy-makers can filter the content of online information the youth encounter and control the time use of the youth, so as to prevent people from lying flat and using digital device excessively.

## Limitations and future directions

Our work is not free of limitations. First, although the mentality or subculture similar to lying flat is also popular in other countries, our work only relies on the Chinese samples. The present findings needs to be replicated and extended in other cultures. Second, there is no neutral condition in our Study 2. Although we showed the distinct effect of lying flat and involution, we cannot tell whether it is lying flat decreased or it is involution increased state self-esteem and smartphone use desire. Future work may include a neutral condition to address this issue, when designing the experiment. Third, we only used an agency to capture problematic smartphone use at the state level. Desire is often used to be an index of addiction (Zhu, 2024), but it would be more convincing to explore the causal link between lying flat and problematic smartphone use behavior. Future work may solve this problem using longitudinal and/or experience sampling designs. Fourth, self-esteem emerged as a partial mediator in mediating the correlational and causal link between lying flat and problematic smartphone use, but other psychological constructs such as coping strategies, social support and negative mood may also play mediating roles. Therefore, effort is also needed to test the role of these constructs that have been listed as risk factors leading to problematic smartphone use (Billieux, 2012).

## Conclusion

Rapid social and technological development comes at the cost of exerting highly pressure. People, especially the youth, may choose lying flat when facing such an unprecedented challenge. However, lying flat and do little to compete for resources is also detrimental on self-esteem, resulting in problematic smartphone use. More theories and corresponding measures are needed to prevent people from lying flat and playing on their digital device (e.g., smartphones) excessively.

## Data Availability

The raw data supporting the conclusions of this article will be made available by the authors, without undue reservation.

## Footnotes

^1^ https://osf.io/6yru9/?view_only=2a8f13bbdf404f9fa601cc8d378f9d7b

^2^We obtained the Chinese version of Lying Flat Tendency scale from one of the developers (i.e., Feng Kong).

^3^We also tested an alternative model to examine whether primed lying flat decrease state self-esteem through the mediating role of smartphone use desire. However, the fitness of this alternative model was worse than the hypothesized one, *F*(2, 189) = 34.13, R^2^ = 0.27, *p* < 0.001, *SE* = 0.05, indirect effect = −0.18, 95% CI [−0.29, −0.10].
